# Dosimetric accuracy of the Convolution algorithm for Leksell Gamma Plan radiosurgery treatment planning: Evaluation in the presence of clinically relevant inhomogeneities

**DOI:** 10.1002/acm2.13903

**Published:** 2023-01-19

**Authors:** Evaggelos Pantelis, Andreas Logothetis, Emmanouil Zoros, Eleftherios P. Pappas, Panagiotis Papagiannis, Ian Paddick, Håkan Nordström, George Kollias, Pantelis Karaiskos

**Affiliations:** ^1^ Medical Physics Laboratory Medical School National and Kapodistrian University of Athens Athens Greece; ^2^ Medical Genesis Care Centre for Radiotherapy Cromwell Hospital London UK; ^3^ Elekta Instrument AB Stockholm Sweden; ^4^ Departments of Medical Physics and Gamma Knife Hygeia Hospital Athens Greece

**Keywords:** bone inhomogeneity, dose calculation accuracy, dose to medium, Gamma Plan Convolution, Leksell Gamma Knife

## Abstract

**Purpose:**

The Leksell Gamma Plan Convolution algorithm (LGP‐Convolution) has not been widely adopted. This mainly stems from the higher calculated beam‐on times relative to the standard ray tracing‐based LGP‐TMR10 dose calculation algorithm. This study aims to evaluate the accuracy of the LGP‐Convolution in scenarios where the treated lesions are in the vicinity of or encompassed by bone and/or air inhomogeneities.

**Methods:**

The solid water dosimetry phantom provided by the vendor was modified to include bone and air inhomogeneities. Two treatment planning scenarios were investigated involving a single shot and multiple shots, respectively. Treatment planning and dose prescription were performed using the LGP‐Convolution algorithm. Triple channel film dosimetry was performed using GafChromic EBT3 films calibrated in terms of absorbed dose to water in a ^60^Co beam. Monte Carlo (MC) simulation dosimetry was also performed in the inhomogeneous experimental geometry using the EGSnrc MC platform and a previously validated sector‐based phase‐space source model. MC simulations were also employed to determine correction factors required for converting EBT3 measurements at points within the bone and air inhomogeneities from dose‐to‐water values to the corresponding dose to medium values.

**Results and Conclusions:**

EBT3 dose to medium correction factors ranged with field size (4, 8, or 16 mm) within 0.941–0.946 for bone and 0.745–0.749 for air inhomogeneities. An excellent agreement was found between the LGP‐Convolution calculations with corresponding EBT3 and MC dose to medium results at all measurement points, except those located inside the air inhomogeneity. The latter is of no clinical importance and excluding them yielded gamma index passing rates of nearly 100% for 3% local dose difference and 1 mm distance‐to‐agreement criteria. The excellent agreement observed between LGP‐Convolution calculations and film as well as MC results of dose to medium indicates that the latter is the quantity reported by the LGP‐Convolution.

## 1 INTRODUCTION

The Leksell Gamma Knife^®^ (LGK) (Elekta Instrument AB, Stockholm, Sweden) Stereotactic Radiosurgery system was developed to treat intracranial lesions using multiple non‐coplanar photon beams produced by ^60^Co radioactive sources.^1^, ^2^ Radiosurgery treatment planning for the LGK is performed using the Leksell GammaPlan^®^ (LGP) (Elekta AB, Stockholm, Sweden) dedicated Treatment Planning System (TPS) which offers two dose calculation algorithm options; the TMR10 algorithm based on ray tracing and pre‐calculated off‐axis ratios, and a Convolution algorithm.^2^, ^3^, ^4^ The LGP‐Convolution algorithm is based on collapsed cone convolution methods enabling precise dose estimation in regions with pronounced tissue inhomogeneities (e.g., lesions in the cavernous sinus or the sphenoid sinus anatomical regions).^4^ There are several dosimetry studies comparing the dose distributions obtained with the LGP‐Convolution and the LGP‐TMR calculation algorithms.^5^, ^6^, ^7^, ^8^ These studies showed that Beam‐On‐Times (BOTs) calculated using the LGP‐Convolution are up to 10% greater than those calculated using the LGP‐TMR10 algorithm, for the same shot configuration and prescription doses. The observed differences, which were found to depend on the position of the treated lesions, were attributed to the presence of tissue inhomogeneities.^5^, ^6^, ^7^, ^8^ Apart from the relatively increased BOTs, longer calculation times and a full CT scan of the patient are required for the LGP‐Convolution algorithm. These differences combined with the fact that the entire LGK clinical experience has been established on the basis of the LGP‐TMR algorithm, have led to modest adoption rates of the LGP‐Convolution in routine radiosurgical practice.^2^ This situation might be reversed by additional studies evaluating the benefit of the LGP‐Convolution in terms of dosimetric accuracy in heterogeneous model geometries.

LGK BOT calculations are based on decay corrected dose rate measurements for the largest available collimation, performed at the Unit Center Point (UCP) with coordinates of (x, y, z) = (100, 100, 100) in the Leksell stereotactic coordinate system.^2^ The AAPM TG‐178 suggests that the dosimetric formalism proposed by Alfonso et al.^9^ and followed in the TRS‐483 small field dosimetry Code of Practice (CoP) with minor modifications,^10^ should be clinically applied for the dose rate calibration of LGK units. Applying the formalism of Alfonso et al.,^9^ the LGK unit is calibrated in terms of dose to water at the center of a spherical water phantom of 160 mm diameter positioned at the UCP. Therefore, both the LGP‐TMR10 and LGP‐Convolution dose calculation algorithms are calibrated in dose to water.

While early versions of dose calculation algorithms implemented in TPSs considered the patient composition to be water (of different densities in most cases), many modern algorithms also consider tissue characteristics during calculations.^11^, ^12^ Such consideration is in accordance with the goal of having the TPS accounting for the differences between water and tissue.^12^ Regarding the LGP, the LGP‐TMR10 algorithm ignores the density and atomic composition of the irradiated tissues and treats the whole head as uniform water material.^3^ On the other hand, the LGP‐Convolution accounts for the characteristics of the irradiated tissues, albeit with some ambiguity as to whether dose to medium or dose to water is reported.^4^ The AAPM TG‐329 reported that the dose to water is calculated by the LGP‐Convolution algorithm, citing a white paper published by the vendor.^12^ The medium used to report dose is not however explicitly mentioned in the cited study.^4^ Moreover, two independent, Monte Carlo (MC) based, studies reported excellent agreement between LGP‐Convolution calculations and corresponding MC dose to medium results.^8^, ^13^ Hence the ambiguity lingers, affecting dose prescription and hindering dose verification.

This study aims to evaluate the accuracy of the dose distributions calculated by the LGP‐Convolution in situations where treated lesions are in proximity to bone and air inhomogeneities. To this end, clinically relevant treatment plans were developed containing single or multiple composite shots using the LGP‐Convolution algorithm and delivered to a modified solid water phantom containing bone and air inhomogeneities. GafChromic^TM^ EBT‐3 films (Ashland Inc., Wayne, NJ) and a precise film dosimetry protocol were used for experimental dose verification. Independent dose calculations were also performed for the treatment plans using the EGSnrc MC platform and a previously validated sector‐based phase space source model.^8^

## 2 MATERIALS AND METHODS

### 2.1 The Leksell Gamma Knife and dose rate calibration measurements

All dose calculations and experimental measurements of this study were performed for a LGK Perfexion (LGK‐PFX) unit. The LGK‐PFX unit consists of a fixed collimator that resembles a tapered cylinder whose axis of symmetry is along the longitudinal axis of the treatment couch. The ^60^Co sources are attached to eight moveable plates (called sectors) located around the outer surface of the collimator. Each sector contains 24 sources, giving a total of 192 sources, and the collimator body contains 576 channels to accommodate three field sizes for each of the 192 sources.^2^, ^8^ Each sector can be moved independently along a conical surface to facilitate alignment of the sources with any of the available field sizes labeled as 4, 8, and 16 mm, as well as in the blocked position, allowing the use of composite shots. Moreover, it is noted that the LGK‐PFX model shares the same irradiation unit as the more recent ones LGK‐Icon^TM^ and LGK‐Esprit^TM^. More details on the LGK irradiation units are out of the scope of this study and can be found elsewhere.^2^

The dosimetric formalism by Alfonso et al.^9^ was applied for dose rate calibration measurements in accordance with the TG‐178 report.^2^ The Semiflex 31010 (PTW, Freiburg, Germany) ionization chamber having a sensitive volume 0.125 cm^3^ was used. The chamber was calibrated in terms of absorbed dose to water, N_D,w_, at the ^60^Co gamma ray unit of the Ionizing Radiation Calibration Laboratory (IRCL) of the Greek Atomic Energy Commission (GAEC).† Measurements were performed with the chamber positioned at the center of the Elekta LGK Solid Water (LGK‐SW) spherical phantom^2^ using an appropriate adapter. The specific phantom has a diameter of 160 mm and aligns the chamber at the UCP with its stem along the z‐axis of the system.^2^ Using this orientation none of ^60^Co beams pass through the stem.^14^ The chamber was inserted to the phantom which was attached on the treatment couch and aligned at the UCP. The largest available field size of 16 mm was used to irradiate the chamber for 1 min. The charge created was collected using a PTW UNIDOS electrometer. Five irradiations were performed using the same irradiation time. The average collected charge was calculated and corrected for environmental conditions (i.e., temperature and pressure), polarity and ion‐recombination effects. Since the chamber was calibrated in a ^60^Co gamma ray field the $K_{Q,Q_{o}}$ beam quality correction factor was assumed equal to 1, while a $K_{Q_{msr},Q}^{f_{msr},\;f_{ref}}$ machine specific reference field correction factor of 1.0037 for the PTW‐31010 chamber was used.^2^ A dose rate of 3.215 Gy/min was obtained which agreed within 0.5% with the calibration dose rate of the system, corrected for ^60^Co radioactive decay.

### 2.2 The inhomogeneous phantom

**FIGURE 1:**
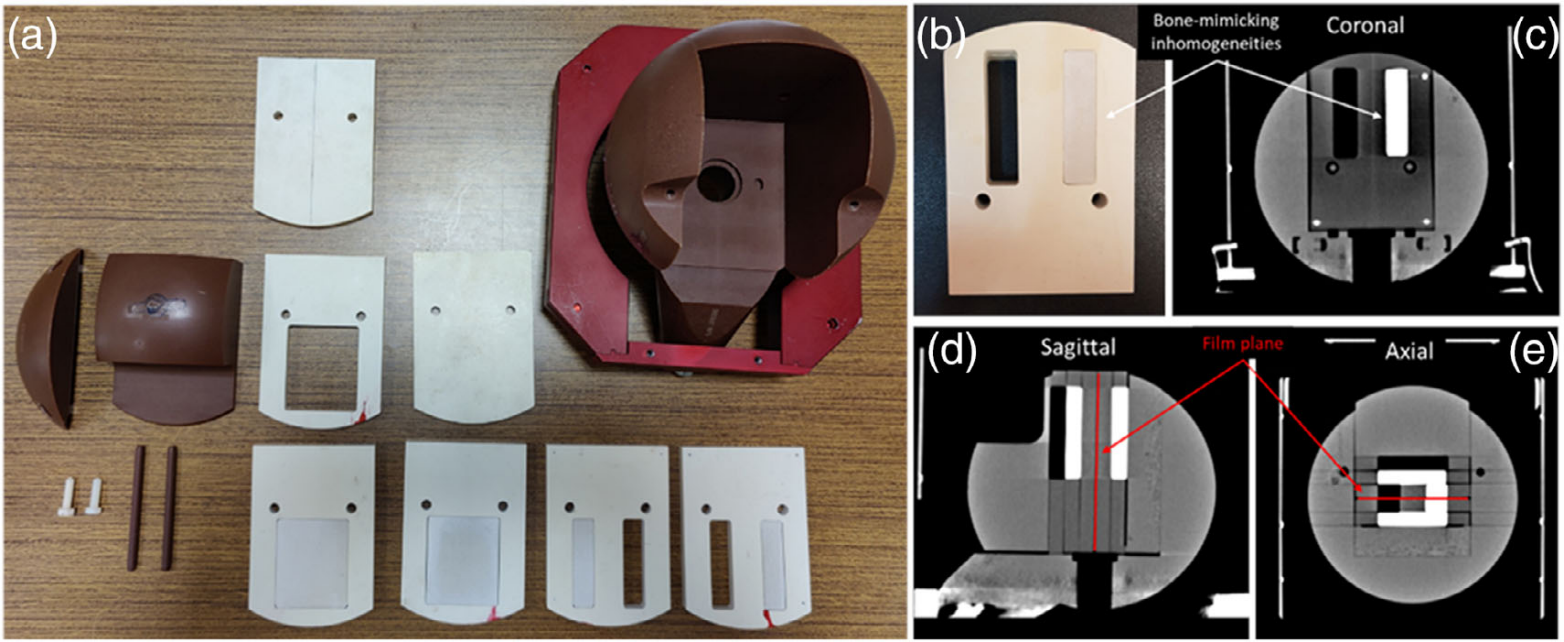
(a,b) The Leksell GK SW phantom (shown with brown color) and the custom‐made PTW‐RW3 white colored slabs incorporating the 3D‐printed bone‐mimicking inhomogeneity. (c)‐(e) Coronal, sagittal, and axial CT slices of the phantom. The location of the film piece is presented in subfigure (c) at coronal orientation and depicted by the red lines at sagittal and axial orientations. The three metal pins embedded at the periphery of the RW3 slab holding the film are also shown.

The LGK‐SW phantom used for calibration dose rate measurements was modified by replacing the five central solid water slabs with corresponding custom‐made PTW‐RW3 slabs of the same dimensions. The RW3 slabs incorporated rectangular parallelepiped bone and air inhomogeneities (Figure 1a). When the RW3 slabs were mounted in the SW phantom, the bone inhomogeneity had a mirrored C shape in the axial plane (Figure 1e) with a length of 6 cm along the z‐axis (craniocaudal axis) and a thickness of 1 and 1.5 cm along the y‐axis (anteroposterior axis) and x‐axis (left‐right axis), respectively (Figure 1c,d). The bone inhomogeneity partially surrounded a homogeneous RW3 region with dimensions of (Δx, Δy, Δz) = (2 × 2 × 6) cm^3^, which was further enclosed by an air rectangular parallelepiped cavity of (Δx, Δy, Δz) = (1.5 × 2 × 6) cm^3^ in size. Three metal pins were also placed at the edges of the central RW3 slab (visible in Figure 1c). Their purpose was to lance through the GafChromic film to facilitate the spatial co‐registration procedure (see Section 2.4).

The LGK‐SW phantom with the inhomogeneous slabs loaded, was CT scanned using a SOMATOM Confidence CT scanner (Siemens Healthcare GmbH, Erlangen, Germany) and the following scanning parameters: 120 kVp, 1 s tube rotation, 300 mA, 0.5 pitch, 512 × 512 reconstruction matrix, 1 mm reconstructed slice thickness, and 27.3 cm field of view (FOV) resulting in voxel dimensions of (0.53 × 0.53 × 1) mm^3^.

The nominal mass density and relative electron density values of the materials used to construct the inhomogeneous phantom are presented in Table 1. Bone inhomogeneities were 3D printed using the Project 360 (3D Systems, Morrisville, NC) printer and a calcium‐based raw material (RTsafe P.C., Athens, Greece). The mass density of the printed bone material has been found similar to that of human skull yielding a Hounsfield Unit (HU) agreement to within 1%.^15^ More details of the procedure followed to print the bone inhomogeneity can be found elshewere.^15^ The average mass density and relative electron density values of the materials comprising the phantom were also extracted from the phantom's CT images using the HU to mass density and relative electron density calibration data of the CT scanner employed. The calculated values are presented in Table 1, where an agreement within uncertainties with the corresponding nominal values can be observed for all materials except for the mass density of the RW3. Since, the LGP‐Convolution and MC based dose calculations algorithms obtain the measured HU to density values on a voxel by voxel basis using the HU to density calibration data of the CT scanner (see Sections 2.3 and 2.5), the observed mass density difference for the RW3 material relative to its nominal value does not affect the dosimetry results of this study.

**TABLE 1:** Nominal and average measured mass and relative electron densities (if available) of the materials comprising the inhomogeneous phantom. The corresponding mass density values calculated using Equation (1) based on the measured relative electron densities are also shown.

	**Mass density, ρ (g/cm^3^)**			**Relative electron density,** η	
**Material**	**Nominal**	**Measured**	**LGP‐Convolution**	**Nominal**	**Measured**
RW3	1.045	1.014 ± 0.010	0.993	1.012	0.993 ± 0.019
LGK‐SW	1.043	1.038 ± 0.010	1.015	1.011	1.013 ± 0.019
Bone mimicking	1.709^a^	1.720 ± 0.045	1.711	‐	1.605 ± 0.054

^a^ The nominal density of the bone mimicking material has been taken from Ref. 15.

### 2.3 Treatment planning using the LGP‐Convolution algorithm

The LGP‐Convolution algorithm is based on Collapsed Cone (CC) convolution techniques^16^ to calculate the absorbed dose at each voxel comprising the patient model geometry. The total dose deposited in each voxel is calculated using a primary‐scatter separation technique according to which, the dose from primary photons (contributing to more than 90% of the total dose close to each beam) and from higher‐order scattered photons (contributing to the dose away from beam axis) is calculated separately and summed.^4^ The TERMA is calculated by scaling corresponding MC pre‐calculated data for a 160 mm diameter water sphere aligned at the UCP, using relative electron density and mass density voxel values. The primary and scatter kernels have also been precalculated using MC methods and are scaled with the radiological path length during dose calculations.

The HUs from patient's CT images are mapped to relative electron densities (η) using the HU to relative electron density calibration data of the CT scanner on a voxel‐by‐voxel basis. The mass density (ρ) of each voxel is calculated using the equation^4^:

(1) $$\rho \;=\left\{\begin{array}{c} \eta \;\;\;\;\;,\eta \;\leq 1\\ \frac{1}{0.85}\left(\eta -0.15\right),\;\eta >1\; \end{array}\right.$$

The mass density values of the materials used to construct the inhomogeneous phantom were calculated using Equation (1) and the corresponding average relative electron densities values were obtained from the phantom's CT images. These density values are also given in Table 1 and are indicative of the values used by the LGP‐Convolution to account for tissue inhomogeneities during treatment planning dose calculations.

**FIGURE 2:**
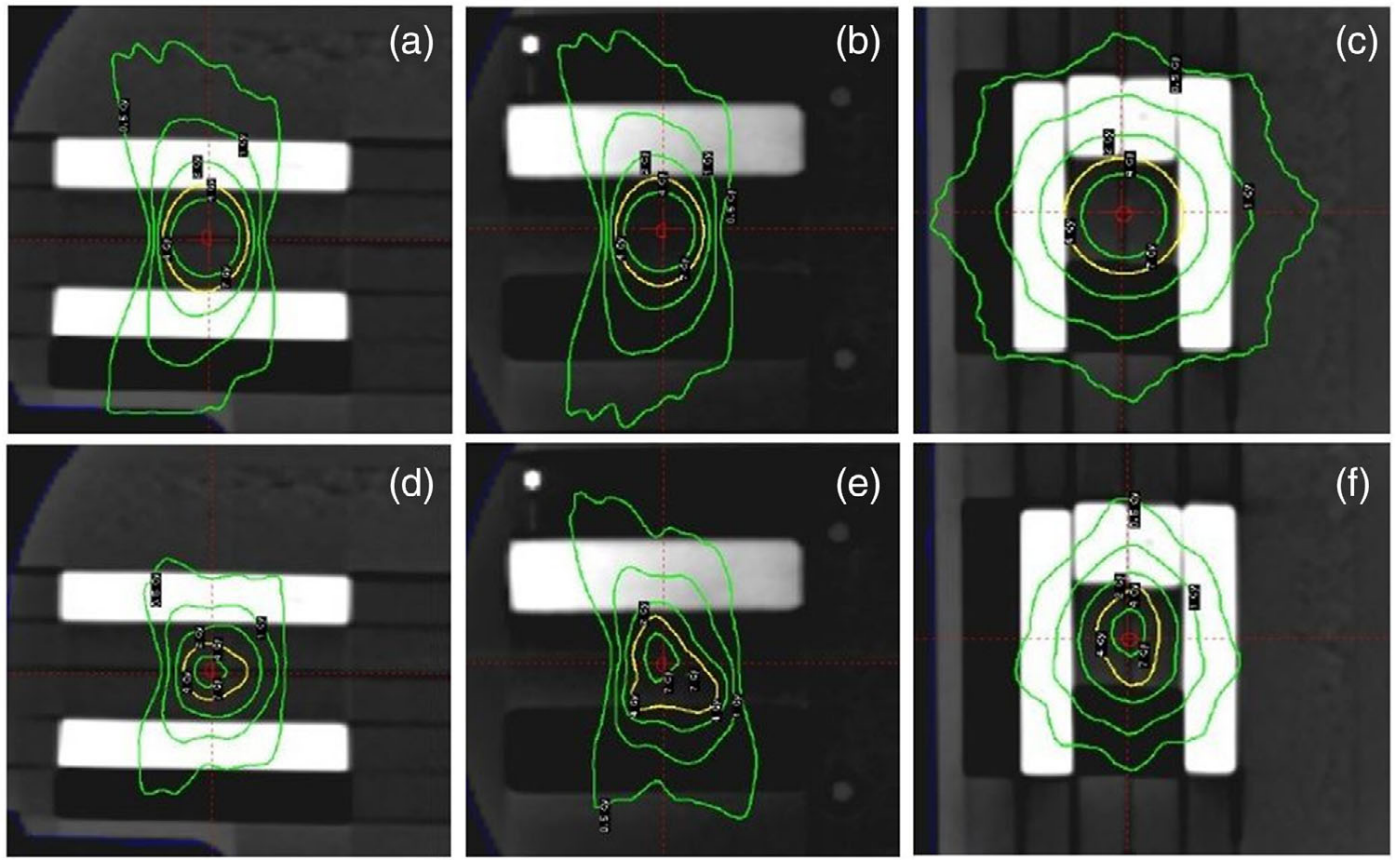
Sagittal, coronal, and axial views of the single shot (a‐c) and the multiple composite shot (d‐f) treatment plans, respectively. The prescribed dose isolines are depicted in yellow color.

The CT images of the inhomogeneous phantom were imported to the LGP v. 11.1.1 TPS. Two treatment plans were developed using the LGP‐Convolution algorithm (Figure 2). The first plan involved a single 16 mm shot located at the RW3 region at the center of the mirrored C shape bone heterogeneity (Figure 2a‐c). A dose of 4 Gy was prescribed at 50% isodose surface which corresponded to a BOT of 3.3 min. A target of an irregular shape was assumed for the second plan. Seven shots were used to irradiate this target. Four of them used the 4 mm field, one used the 8 mm field, while the rest two shots used sectors aligned with the 4, 8, and 16 mm collimation channels (i.e., composite shots). A dose of 4 Gy was prescribed at the 50% isodose surface which corresponded to a BOT of 12.7 min. Both treatment plans were designed so that: (a) the 50% isodose surface (which commonly represents the delineated target) abuts on the air and bone inhomogeneities (Figure 2) to simulate clinical scenarios where the target is proximal to the skull or the nasal cavity, and (b) most of the beams (if not all) pass through at least one inhomogeneous region prior reaching the simulated target. Dose values for each treatment plan were exported in DICOM RT file format using a dose grid resolution of (1 × 1 × 1) mm^3^ and used for the evaluation of the dosimetric accuracy of the LGP‐Convolution algorithm.

### 2.4 Experimental measurements

GafChromic EBT3 films were used for the evaluation of the dosimetric accuracy of the LGP‐Convolution algorithm. The films were calibrated in terms of absorbed dose to water at the ^60^Co gamma ray unit of the IRCL facilities (see Section 2.1). Calibration EBT3 film pieces with dimensions of (3 × 3) cm^2^ were situated sequentially 5 cm beneath the surface of a 20 cm thick RW3 solid water phantom and irradiated with a (10 × 10) cm^2^ photon field. Calibration doses ranging from 0.5 to 15 Gy were delivered to calibration film pieces. Film pieces were cut to fit into the custom‐made film slab (Figure 1a,b). Each film piece was handled according to the recommendations of the TG‐235 report.^17^ Optical density read‐out of both calibration and experimental films was performed 24 h after irradiation using an EPSON Perfection V850 Pro flatbed scanner in transmission mode disabling all filters and image enhancement options. Film scans were implemented in landscape orientation using a 3 mm thick glass compression plate.^18^ RGB positive images of 48‐bit depth were acquired with a spatial resolution of 150 dpi (i.e., 0.169 mm/px) and saved in tagged image file format. In‐house software routines developed in MATLAB^®^ (The MathWorks, Inc., Natick, MA) were used to read the measured pixel values of each experimental film piece and convert them into dose values using a triple channel technique and the calibration data of the used film batch.^19^

Since the film dosimetry protocol followed yields dose to water within the medium where the films lay (i.e., bone and air),^20^ appropriate correction factors were calculated to convert this to corresponding dose to medium values, according to^20^:

(2) $$K_{med}=\;{\left(\frac{D_{med}}{D_{film}}\right)}^{LGK}{\left(\frac{D_{film}}{D_{w}}\right)}^{Cal}$$

where, ${\left(\frac{D_{med}}{D_{film}}\right)}^{LGK}$is the ratio of dose to medium to dose to film for the LGK photon fields and irradiation geometries and$\;{\left(\frac{D_{film}}{D_{w}}\right)}^{Cal}$ is the ratio of dose to film to dose to water for the film calibration photon field (i.e., the ^60^Co gamma ray reference field) and calibration geometries (see also Section 2.5).

The measured 2D dose to water maps were converted to dose to medium maps and spatially co‐registered with the corresponding TPS calculated 3D dose grid using the three metal pins used to hold the film piece inside the inhomogeneous SW phantom (Figure 1a,b). A rigid transformation matrix was calculated for each film using the geometric centroid of each film hole visible in the scanned images as reference points and the mass centroid of the corresponding metal pins visible in CT images as moving points (Figure 1c). The calculated transformation matrix was used to align the TPS dosimetry data with film measurements.

### 2.5 Monte Carlo calculations

The accuracy of the LGP‐Convolution dose distributions was also evaluated through comparison to MC calculations using EGSnrc.^21^ For this purpose, the treatment plan information was exported from the LGP in XML format. The parsing of plan information, the creation of the computational geometry model, and the generation of the EGSnrc input file, were automatically performed using in‐house software routines developed in MATLAB. A voxelized simulation geometry was created by processing the DICOM CT image set of the phantom. HUs were mapped to mass densities using the HU to mass density calibration data of the used CT scanner on a voxel‐by voxel basis. The atomic composition of each voxel was determined by its density using human tissue composition data published by Schneider et al.^22^ The fiducial box was excluded from the simulation geometry. For each plan, the voxel size was identical to that of the CT (i.e., (0.53 × 0.53 × 1) mm3) in a region encompassing all shot centers with a minimum 30 mm margin, which also served as the dose scoring region. A lower resolution was used for the rest of the geometry, through down sampling by a factor of 8 in the *x* and *y* dimensions.

A sector‐based phase space (phsp) source model developed in‐house and validated in a previous study was used herein.^8^ The advantage of this sector‐based phsp source model is that it allows for the simulation of treatment plans with multiple single and/or composite shots in a single simulation. In more detail, all shots are simulated at the same time and photons are sampled from each sector, rotated, and translated accordingly (which includes photon position and direction rotation), using the probability distribution described in Equation (3):

(3) $$P_{i}=\;\frac{t_{i}N_{i}}{\sum_{i\;=\;1}^{n_{s}}t_{i}N_{i}}$$

(where, $t_{i}$ is the time that sector *i* was active, $N_{i}$ the number of particles stored in phase space file of the collimator of sector *i*, and $n_{s}$ the total number of sectors in the plan) given that the original histories simulated to generate the phase space files were the same for all collimators.

The general purpose “egs_app” user code of the EGSnrc platform was used to calculate the dose at each voxel. MC calculated dose values per emitted fluence were converted to absolute dose values using a scaling factor, F of:

(4) $$F\;=\;\frac{{\dot{D}}^{\mathrm{ref}}}{D_{w,\;16\mathrm{mm}\;}^{\mathrm{MC}}}\cdot [\mathrm{BOT}]$$

where ${\dot{D}}^{\mathrm{ref}}$ is the measured reference dose rate of the used GK unit taking into account the exponential decay of the ^60^Co sources until the plan date, $D_{w,\;16\mathrm{mm}\;}^{\mathrm{MC}}$ is the MC calculated dose to a small (0.5 × 0.5 × 0.5) mm^3^ voxel at the center of a homogeneous water phantom of 160 mm in diameter centered at the UCP and irradiated with the 16 mm field size (see Section 2.1), and BOT is the Beam‐On‐Time of the treatment plan determined using the LGP‐Convolution algorithm.

MC simulations were also performed to determine the dosimetric quantities involved in Equation (2) and calculate the correction factors required to convert the film measurements of dose to water to dose to medium (see Section 2.4). In more detail, the dose to film and dose to water values were calculated for the film calibration, while the dose to film and dose to medium values were calculated for the LGK‐PFX irradiation geometries. For the film calibration geometry, the ^60^Co unit was modeled as a point source located 95 cm away from a cubic water phantom of 30 cm side, emitting photons with energy sampled from the ^60^Co photon energy spectrum published by Mora et al.^23^ The photon beam was geometrically collimated to a field size of (10 × 10) cm^2^ defined at the surface of the phantom. Two simulations were performed for this geometry; one to determine the dose to a small water voxel of (1 × 1 × 0.5) mm^3^ situated along beam axis at 5 cm depth inside the phantom and one to determine the dose to film assuming an (4 × 4) cm^2^ EBT3 film piece aligned perpendicularly to beam axis at the same depth and a scoring voxel of (10 × 10 × 0.025) mm^3^ at the active material of the film. For the simulations in the LGK‐PFX system, a single shot with the 16 mm field size was simulated irradiating the LGK‐SW phantom aligned at the UCP. A bone sphere of 2 cm diameter was modeled at the center of the phantom. Two simulations were performed; one to determine the dose to bone medium using a scoring voxel of 0.5 mm in diameter at the center of the spherical bone inhomogeneity and one to determine the dose to film at the same position assuming a (0.5 × 0.5) cm^2^ film piece and scoring the dose at its active material. Simulations for the LGK‐PFX irradiation geometry were repeated for the air inhomogeneity as well as for the 8 and 4 mm field sizes. The atomic composition and mass density of EBT3 film was taken from the work of Bekerat et al.^24^

Details regarding MC simulation parameters (i.e., cross sections, electron transport parameters, energy cut off values, etc.) are presented in Table 2 following the recommendations of APPM TG‐268.^25^

**TABLE 2:** Summary of the EGSnrc simulation parameters used in this study according to the recommendations of AAPM report TG‐128

**Item Name**	**Description**	**References**
Code, version/release date	EGSnrc, 2022 version (“egs_app” general purpose, bare egs++ application)	^21^
Validation	‐	^26^, ^28^
Hardware & Timing	Simulations were performed in parallel mode on the ARIS high performance computing system 2000‐5000 core hours were required for each simulation	^a^
Source description	Phase space files.	^8^
Cross‐sections	XCOM photon cross sections	^27^
Transport parameters	Electron step algorithm = EGSnrc/PRESTA‐II ECUT = AE = 521 keV PCUT = AP = 1 keV All other parameters were set to default	^21^, ^28^
VRT	None	‐
Scored Quantities	Absorbed dose	‐
# histories/statistical uncertainty	0.5x10^8^ ‐ 4x10^10^ histories depending on the simulations Statistical uncertainties are given in Table 3.	‐
Statistical methods	Batch method	^21^
Post‐processing	‐	

^a^ Details on ARIS HPC can be found at: https://hpc.grnet.gr/en/

### 2.6 Uncertainty budget

Uncertainties were estimated according to the Guide to the expression of Uncertainty in Measurement (GUM).^29^ A detailed uncertainty budget for the GK calibration dose rate measurements, film dosimetry and MC simulations is presented in Table 3 using a level of confidence of 68% (k = 1). Regarding the calibration dose rate, an uncertainty of 0.9% was estimated taking into account the statistical uncertainty of the collected charge calculated as the standard deviation of the mean, the uncertainty ascribed to the charge measurement influence quantities (i.e., K_TP_, K_pol_, K_ion_),^30^ the uncertainty of the chamber $K_{Q_{msr},Q}^{f_{msr},\;f_{ref}}$ correction factor for the LGK msr field (i.e., 16 mm cone)^2^ and the uncertainty of the chamber's calibration coefficient (N_D,w_). An uncertainty of 2% was ascribed to film dosimetry results calculated based on the uncertainty of the calibration data, the uncertainty of OD measurements, the homogeneity of the optical scanner^31^ and the uncertainty of the dose to medium correction factor (K_med_). Since, K_med_ is calculated as ratios of MC determined dose values (see Equation 2), only the corresponding statistical uncertainties of the used dose values were considered. An uncertainty of 1.4% was estimated for the reported MC simulation dose results. This was calculated from the corresponding statistical uncertainty and the uncertainties ascribed to the scaling factor used to convert MC values per emitted fluence to absolute dose values (see Section 2.5 and Equation 4). Finally, the use of metal pins for spatially registering the film dose results with the corresponding LGP dose data is related to a spatial registration uncertainty of 0.5 mm (i.e., equal to the half of the slice thickness of the CT images of the phantom).

**TABLE 3:** Estimated uncertainties associated with the GK calibration, film and MC dose results expressed at 68% confidence level (k = 1). Uncertainty values were obtained according to the Guide to the expression of Uncertainty in Measurement (GUM).^29^

		**Dosimetric uncertainty (%)**		
**Procedure**	**Source of uncertainty**	**Type A**	**Type B**	**Reference**
GK calibration	Collected charge	0.1		‐
	Correction for influence quantities (k_TP_, K_ion_, K_pol_)		0.3	^30^
	Chamber calibration coefficient, N_D,w_		0.7	‐
	Chamber correction $K_{Q_{msr},Q}^{f_{msr},\;\;f_{ref}}$ for the LGK msr field		0.5	^2^
	**Standard uncertainty (combined type A and B)**	**0.9**		
Film dosimetry	Calibration data^a^		1.9	‐
	OD measurement reproducibility	0.3		‐
	Optical scanner homogeneity		0.2	^31^
	Dose to medium correction factor, K_med_		0.3	‐
	** Standard uncertainty (combined type A and B)**	**2.0**		
MC calculations	Scaling factor, F (see Equation 4)	0.15	0.9	‐
	$D_{med}^{LGK},\;\;D_{film}^{LGK},\;\;D_{w}^{Cal}$, $\;D_{film}^{Cal}$ for calculating the dose to medium correction factor (see Equation 2)	0.15	0.78	^26^
	$D_{med}^{LGK}$, for the simulated treatment plans	0.5	0.78	^26^
	** Standard uncertainty (combined type A and B)**	**1.4**		

^a^ The uncertainty ascribed to the film dosimetry calibration data includes the uncertainty of the calibration fit parameters (1.8%) and the uncertainty of the delivered dose values (0.6%).

The bold values are used to depict the combined standard uncertainty of each procedure.

### 2.7 LGP‐Convolution dosimetric accuracy analysis

The accuracy of LGP‐Convolution dose calculations for both treatment plans was evaluated by comparing the TPS dose values with corresponding film and MC dose to medium results. Comparison was performed in terms of 1D dose profiles, as well as of dose maps using three‐dimensional (3D) gamma index (GI) analysis and 3% local Dose Difference (DD%) and 1 mm Distance‐to‐Agreement (DTA) passing criteria, taking into account the TG‐178 reccomendations^2^ and the uncertainty budget of the reported dose results (Table 3). Film and MC dose results were resampled to an ultra‐fine spatial grid with a resolution of 0.1 mm prior to comparison and used as the reference dose distributions in the conducted GI analysis.^32^ TPS calculations always served as the evaluated dose distribution. Although voxels within air are of minimal clinical importance, gamma analyses were performed with and without considering these voxels.

## 3 RESULTS

### 3.1 Dose to medium correction factors

Table 4 summarizes the correction factors used for converting film measurements to dose to medium within the bone and air inhomogeneities of the phantom, for the three available field sizes of the LGK‐PFX system. A small dependence on field size can be observed which is however within statistical uncertainty. Average correction factors of 0.944 and 0.747 for points located inside the bone and air inhomogeneities, respectively, were used for the multiple shot treatment plan. For the 16 mm single shot treatment plan, the corresponding correction factors in Table 4 were used for correcting the film measured dose values in the bone and air inhomogeneities.

**TABLE 4:** K_med_ correction factors for converting the measured film dose to water values to dose to medium values for the bone and air inhomogeneities of the inhomogeneous phantom and for the three field sizes available in the LGK‐PFX system

**Field size (mm)**	$K_{med}$ **correction factor**	
	**bone**	**air**
16	0.941 ± 0.003	0.745 ± 0.002
8	0.946 ± 0.003	0.748 ± 0.003
4	0.946 ± 0.004	0.749 ± 0.003

### 3.2 LGP‐Convolution dosimetric accuracy

#### 3.2.1 Single shot plan

**FIGURE 3:**
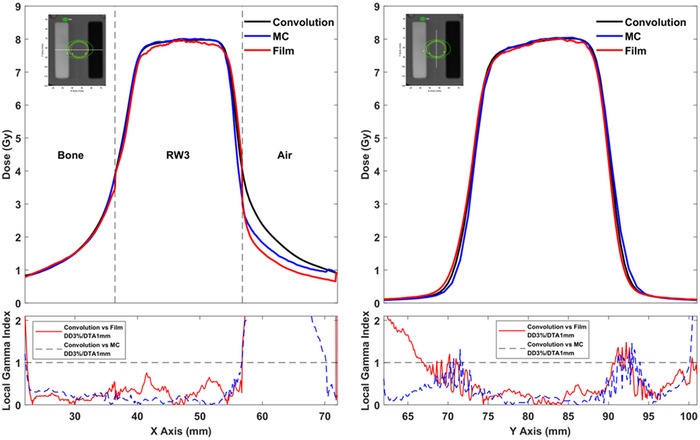
LGP‐Convolution, EBT3 and MC dose profile data along the x‐axis (left) and y‐axis (right) for the single shot treatment plan. The lines along which dose profile data were retrieved are depicted at the reconstructed CT slices of the slabs incorporating the film in the inset of each plot. The TPS‐calculated dose isolines are also superimposed on the presented axial slice. Corresponding 1D gamma index results calculated using 3%/1 mm local DD%/DTA criteria are also presented beneath each profile plot.

In Figure 3, EBT3 measured, as well as corresponding MC and LGP‐Convolution calculated dose profile data for the single shot treatment plan, are plotted along the x and y axes of the stereotactic space. The inset of each plot depicts the axial CT slice of the inhomogeneous phantom and the line along which dose profile data were retrieved. As evidently shown in the y‐axis dose profile plot, which does not involve inhomogeneous regions, the LGP‐Convolution dose values are in excellent agreement with corresponding EBT3 and MC results. Ignoring the points laying inside the air inhomogeneity, an excellent agreement between the LGP‐Convolution dose values with corresponding EBT3 and MC results can also be observed in the x‐axis dose profile plot crossing the bone and air inhomogeneities of the phantom. This agreement is maintained also at points laying very close (within 1 mm) to the interface between RW3 and the bone or air inhomogeneities. Significant differences can be observed only at points lying inside the air inhomogeneity. At these points of reduced clinical importance, the LGP‐Convolution significantly overestimates dose relative to corresponding EBT3 and MC results. Gamma index analysis of the presented dose profile data sets was performed for a quantitative evaluation of the LGP‐Convolution accuracy. GI results using local DD%/DTA criteria of 3%/1 mm are also plotted in Figure 3 confirming the excellent agreement between the LGP‐Convolution dose profile data with EBT3 and MC results at all points except those within the air inhomogeneity.

**FIGURE 4:**
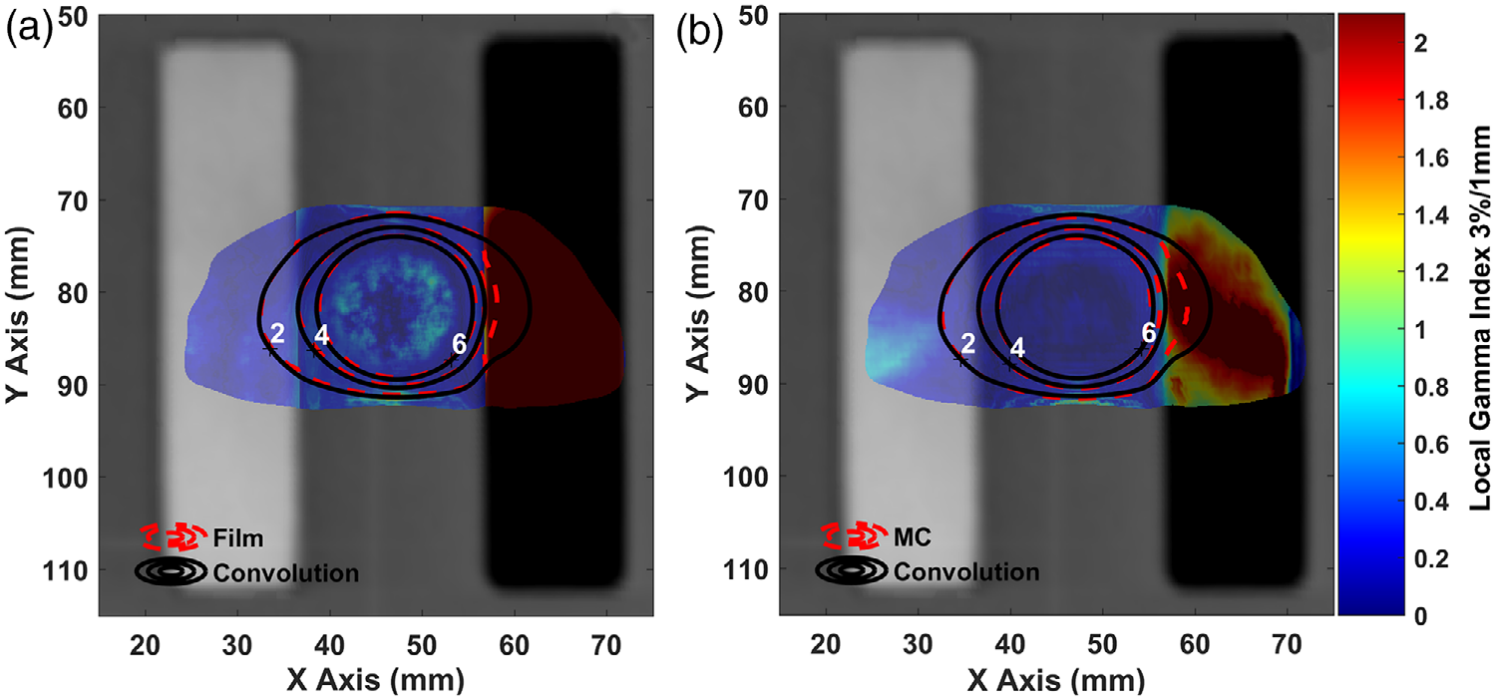
EBT3 film (a) and MC (b) dosimetry results, as well as corresponding LGP‐Convolution dose data for the single shot treatment plan, plotted in terms of isodose lines superimposed on the axial CT slice of the inhomogeneous phantom. The gamma index maps calculated using 3%/1 mm local DD%/DTA criteria, for the evaluation of the LGP‐Convolution dose calculation accuracy in inhomogeneous geometries, using corresponding EBT3 (a) or MC (b) results as reference are also superimposed using an RGB color scale. A dose cut‐off threshold of 1 Gy has been applied.

In Figure 4, the dose distribution of the single shot treatment plan obtained by the LGP‐Convolution algorithm is presented in terms of 2D isodose lines along with corresponding EBT3 and MC results. The presented results confirm the excellent agreement between EBT3 and MC results with the LGP‐Convolution calculated dose values for all pixels lying in the homogeneous and bone inhomogeneity areas. For points laying inside the air inhomogeneity the LGP‐Convolution algorithm clearly overestimates the delivered dose. Corresponding GI passing rates are given in Table 5, revealing that excluding the points laying inside the air inhomogeneity, nearly 100% of the LGP‐Convolution evaluated dose values pass the 3%/1 mm DD%/DTA criteria. When these points of reduced clinical importance are included in the analysis, the passing rates drop to 71.5% and 79.8%, using the EBT3 and MC as reference dose distributions, respectively.

**TABLE 5:** Gamma index passing rates for the evaluation of the dose calculation accuracy of the LGP‐Convolution algorithm in inhomogeneous model geometries. GI analysis was performed using 3%/1 mm local DD%/DTA criteria and the EBT3 or the MC results as the reference dose distribution and the LGP‐Convolution as the evaluated distribution. A dose cut‐off threshold of 1 Gy has been applied to all calculations. Passing rates for each treatment plan were calculated both considering and ignoring the points within the air inhomogeneity

	**Gamma Index passing rates (%)**			
	**Single shot plan**		**Multiple shot plan**	
**Reference dose distribution**	**Excluding air**	**Including air**	**Excluding air**	**Including air**
EBT3	99.8	71.5	99.8	73.3
MC	99.9	79.8	99.9	81.0

#### 3.2.2 Multiple shot plan

**FIGURE 5:**
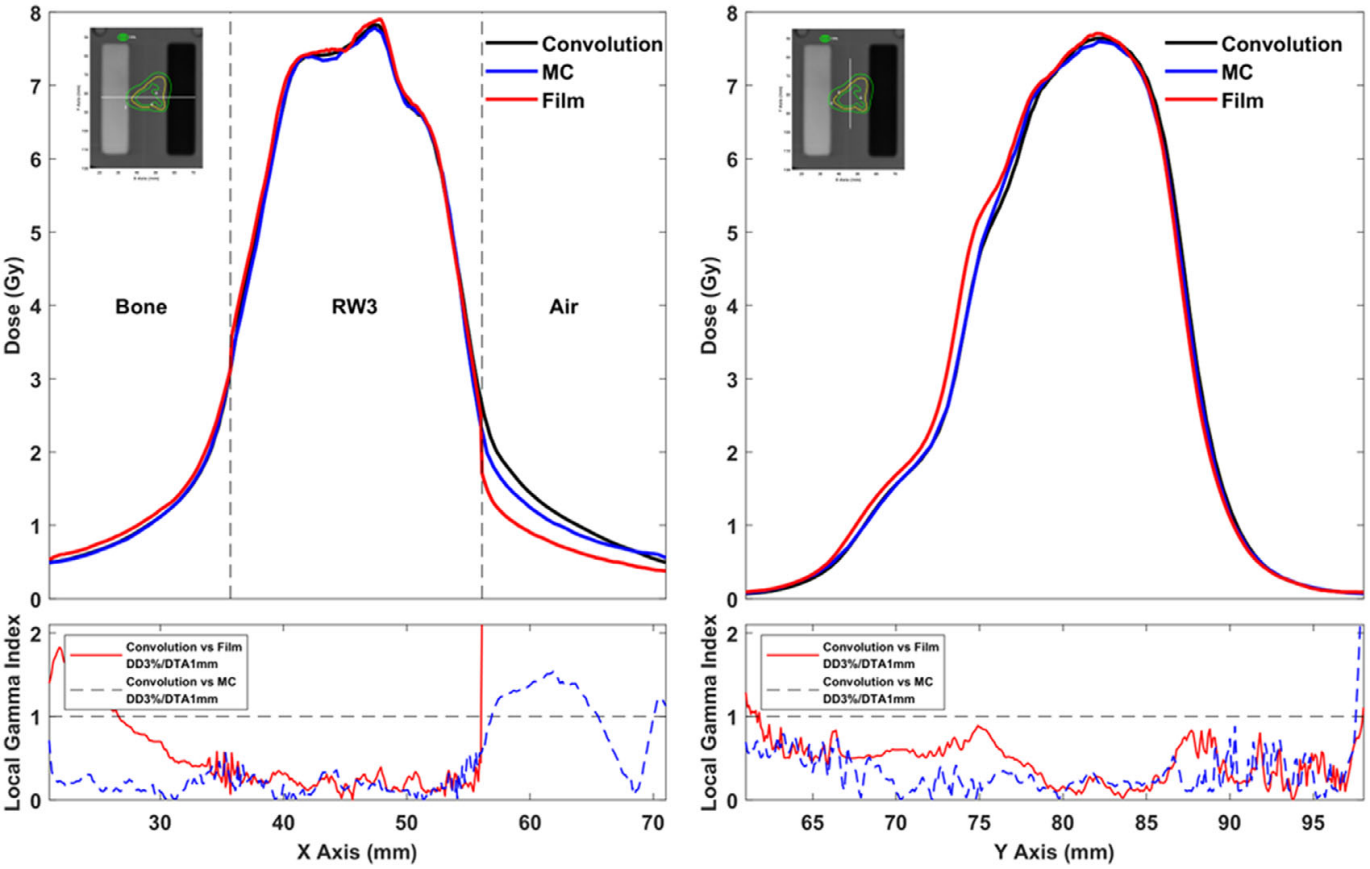
LGP‐Convolution, EBT3 and MC dose profile data along the x‐axis (left) and y‐axis (right) for the multiple shot treatment plan. The lines along which dose profile data were retrieved are depicted at the reconstructed CT slices of the slabs incorporating the film in the inset of each plot. The TPS‐calculated dose isolines are also superimposed on the presented axial slice. Corresponding 1D gamma index results calculated using 3%/1 mm local DD%/DTA criteria are also presented beneath each profile plot.

Figure 5 presents the same data as Figure 4 for the multiple shot treatment plan. A general good agreement can be observed between the three data sets for the dose profile along the y‐axis which does not cross any inhomogeneous regions. The EBT3 film results, however, slightly overestimate dose relative to MC calculated data at y‐axis values ranging from 67 to 77 mm. A good agreement can also be observed for the dose profile along the x‐axis which crosses the bone and air inhomogeneities, at all points except those lying inside the air inhomogeneity. As also observed in the single shot plan, the LGP‐Convolution significantly overestimates the dose within the air inhomogeneity whereas a fair agreement between MC and EBT3 results can be observed. Similar to the results for the single shot plan, the LGP‐Convolution succeeds in predicting the dose delivered at points lying within 1 mm from the interface of RW3 with bone and air inhomogeneities. GI results obtained using 3%/1 mm DD%/DTA criteria are also plotted in Figure 5 confirming the good agreement between the LGP‐Convolution dose profile data with EBT3 and MC results at all points except those lying within the air inhomogeneity.

**FIGURE 6:**
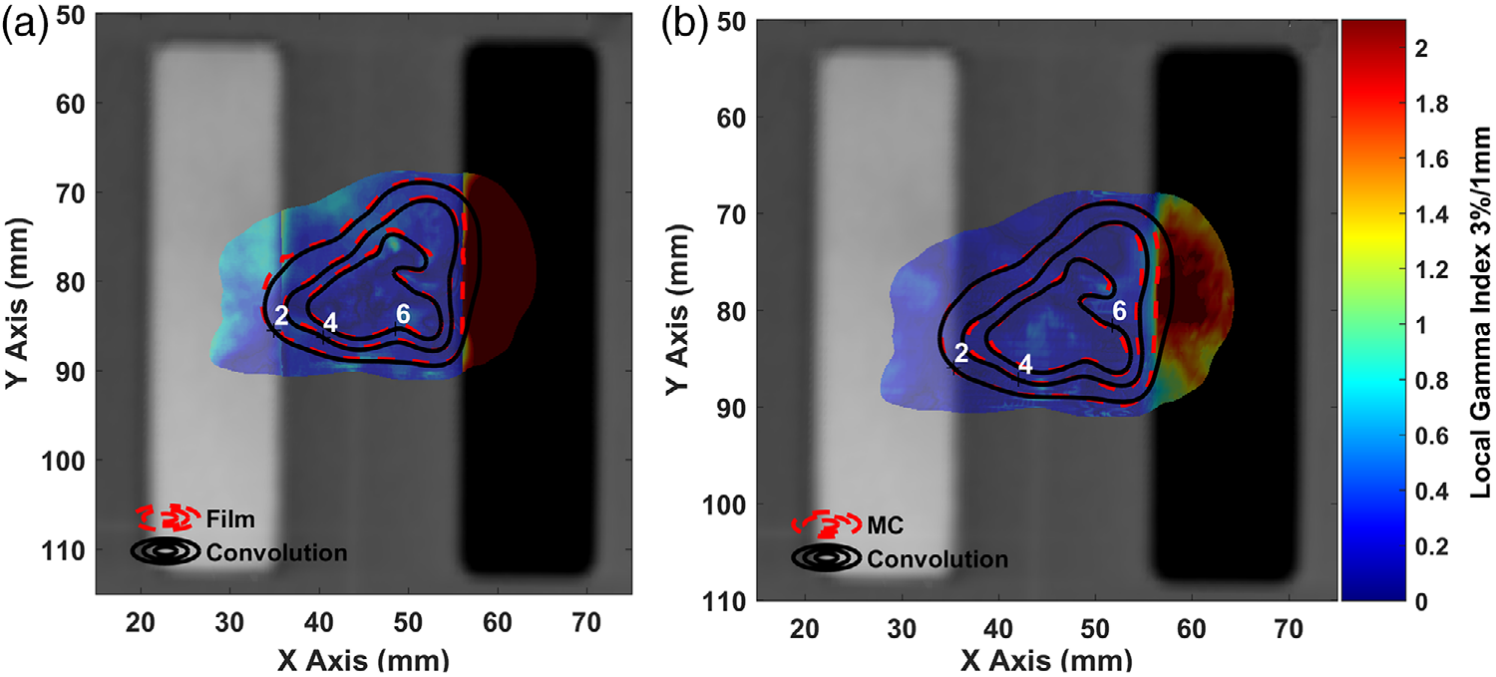
EBT3 film (a) and MC (b) dosimetry results, as well as corresponding LGP‐Convolution dose data for the multiple shot treatment plan, plotted in terms of isodose lines superimposed on the axial CT slice of the inhomogeneous phantom. The gamma index maps calculated using 3%/1 mm local DD%/DTA criteria, for the evaluation of the LGP‐Convolution dose calculation accuracy in inhomogeneous geometries, using corresponding EBT3 (a) or MC (b) results as reference are also superimposed using an RGB color scale. A dose cut‐off threshold of 1 Gy has been applied.

In Figure 6, the dose distribution of the multiple shot treatment plan obtained by the LGP‐Convolution algorithm is presented in terms of 2D isodose lines along with corresponding EBT3 and MC results. The presented results confirm the excellent agreement between EBT3 and MC results with the LGP‐Convolution calculated dose values for all pixels lying in the homogeneous and bone inhomogeneity areas. At points within the air inhomogeneity however, the LGP‐Convolution algorithm clearly overestimates the delivered dose values. Corresponding GI passing rates reveal that excluding points lying inside the air inhomogeneity, nearly 100% of the LGP‐Convolution evaluated dose values pass the 3%/1 mm DD/DTA criteria (see Table 5). When the points inside the air inhomogeneity are included in the analysis, the passing rates drop to 73.3% and 81%, using the EBT3 and MC as reference dose distributions, respectively.

## 4 DISCUSSION

Comparison with EBT3 experimental and MC simulation results for two treatment plans (single 16 mm shot and multiple shots) in an LGK‐SW phantom containing skull bone mimicking and air inhomogeneities, showed that the LGP‐Convolution algorithm succeeds in predicting the delivered dose values at all points within the 13% (1 Gy) isodose line except those lying inside the air inhomogeneity. Excluding points inside the air inhomogeneity yielded gamma index passing rates of nearly 100% for 3%/1 mm local DD%/DTA criteria and either EBT3 or MC results as reference dose distributions. This excellent agreement also holds for voxels lying within 1 mm from the interface of RW3 with bone or air inhomogeneities. These findings indicate that the LGP‐Convolution algorithm accurately calculates the BOTs in clinical conditions where targets are located near the skull, nasal cavities, or cavernous sinus. Moreover, given its increased accuracy relative to the TMR algorithm,^8^, ^13^ the LGP‐Convolution should be used for treatment planning in cases where an organ at risk (OAR) is surrounded by a bone inhomogeneity (e.g., the cochlea in acoustic neurinoma treatments). At points lying inside the air inhomogeneity electron equilibrium conditions are not met and therefore the LGP‐Convolution fails to predict the absorbed dose. These points, however, are of no clinical significance.

Several studies have investigated the dosimetric accuracy of the LGP‐Convolution algorithm using MC simulation and experimental dose measurements in both homogeneous and heterogeneous phantom geometries.^8^, ^13^, ^33^, ^34^, ^35^ Focusing on studies in inhomogeneous geometries, Choi et al.^13^ used the Geant4 MC platform along with an in‐house DICOM‐RT toolkit and an anthropomorphic head phantom to show that the LGP‐Convolution algorithm accurately predicts the absorbed dose to medium in treatment scenarios involving lesions located close to the skull or near the sphenoid bone structure. In an experimental study by Dubus et al.,^35^ the STEEV^TM^ anthropomorphic head phantom (CIRS Inc. Norfolkm VA, USA) was used containing aluminum and air inhomogeneities in the vicinity of the central cube used for film dosimetry measurements. The authors found an excellent agreement between LGP‐Convolution calculations and EBT3 film results in the homogeneous part of the phantom and a fair agreement (within 2%) inside the aluminum and air inhomogeneities, albeit in terms of dose to water.

The above results indicate an ambiguity in the literature regarding the dose reporting medium of the LGP‐Convolution algorithm. The convolution based dose calculation algorithms implemented in modern treatment planning systems are able to calculate either dose to medium or dose to water, depending on the specific algorithm implementation details.^12^ With regard to the LGP‐Convolution algorithm, the TG‐329 reports that the dose to water is calculated citing a white paper published by the vendor wherein the dose reporting medium is not however explicitly stated.^12^ The excellent agreement between the LGP‐Convolution dose values with corresponding EBT3 and MC dose to medium results found at points inside the bone inhomogeneity in this study, imply that dose to medium is calculated by the LGP‐Convolution algorithm. This is in agreement with results reported by Choi et al.^13^ using MC simulation. On the other hand, Dubus et al.^35^ assumed that parts of EBT3 film located inside the homogeneous phantom measures dose to water, whereas parts located inside the aluminum and air heterogeneities measure dose to aluminum in aluminum and dose to water in air, respectively. Assuming that the LGP‐Convolution algorithm calculates dose to water, the authors corrected film dose results within the aluminum heterogeneity with the ratio of mass energy absorption coefficients of water to aluminum, following the methodology suggested by Reynaert et al.^36^ This methodology, however, has been proposed for converting TPS calculated dose to bone to corresponding dose to water values and not experimentally derived dose data.^36^ Moreover, given that the range of secondary electrons is larger than the thickness of film's active layer (28 μm), collisional stopping power ratios should be used for converting dose to medium to dose to water.

One potential limitation of the results of this study pertains to the subtleties of phantom material segmentation. While MC and LGP‐Convolution dose calculation algorithms used the same CT imaging data as input for creating the phantom model geometry, different methodologies were used to account for the characteristics of the materials used. In more detail, the LGP‐Convolution algorithm accounts for tissue inhomogeneities by stretching pre‐calculated TERMA and energy deposition kernels based on their mass densities and electron densities relative to water. Density values are obtained by mapping the HU to relative electron density values using corresponding CT calibration data given by the user. Corresponding mass density values are calculated using Equation (1). The atomic composition of the tissues involved are not explicitly accounted for. The employed MC algorithm on the other hand, mapped the HU to mass density value on a voxel‐by‐voxel basis using the HU to mass density CT calibration data. The mass density was used to determine the elemental composition of each voxel using human tissue composition data published by Schneider et al.^22^ Average measured and calculated (using Equation 1) mass density values presented in Table 1 show that while practically the same mass density was used by both the LGP‐Convolution and MC dose calculation algorithms to account for the bone inhomogeneity, different density values were used for the RW3 and solid water materials. These differences, however, are of order of 2% and are expected to have a minimal effect on the findings of this study.

## 5 CONCLUSION

GafChromic EBT3 film and MC simulation were used to evaluate the accuracy of the LGP‐Convolution dose calculation algorithm in treatment planning scenarios involving clinically relevant, bone and air inhomogeneities. An overall excellent agreement was found between the LGP‐Convolution dose calculations with corresponding EBT3 and MC dose to medium results at all clinically relevant points. Significant differences were only observed for points lying inside the air inhomogeneity, that are however of no clinical importance. Excluding points within the air inhomogeneity, local GI passing rates of nearly 100% were found using 3%/1 mm DD%/DTA criteria. The findings of this study indicate that the LGP‐Convolution algorithm calculates dose to medium, in agreement with previous MC based studies in the literature.

## AUTHOR CONTRIBUTION

Conception of the study: Evaggelos Pantelis, Pantelis Karaiskos, Eleftherios Pappas. Monte Carlo calculations and data analysis: Andreas Logothetis. Treatment Planning: Ian Paddick, Pantelis Karaiskos, George Kollias. Experimental measurements and data analysis: Emmanouil Zoros. Writing manuscript: Evaggelos Pantelis. Proof Reading: Eleftherios Pappas, Panagiotis Papagiannis, Ian Paddick, Håkan Nordström, Pantelis Karaiskos.

## CONFLICT OF INTEREST

Håkan Nordström is an employee of Elekta AB.

## Footnotes

†
https://eeae.gr/en/services/calibration‐of‐equipment
